# Exploring the risk of hypospadias in children born from mothers living close to a vineyard

**DOI:** 10.1371/journal.pone.0249800

**Published:** 2021-04-15

**Authors:** Pierre Bougnères, Raphael Porcher, Laure Esterle, David Baker, Adrien de la Vaissière, Sofia Meurisse, Sophie Valtat, Anne-Laure Castell, Pierre Mouriquand, Alain-Jacques Valleron

**Affiliations:** 1 Inserm U1195, University Paris Saclay, Le Kremlin Bicêtre, France; 2 Centre of Research Epidemiology and Statistics (CRESS), University Paris V, INSERM U1153, Paris, France; 3 Department of Pediatric Urology, Hôpital Mère-Enfant, Hospices Civils de Lyon and University Lyon 1, Lyon, France; Université Clermont Auvergne - Faculté de Biologie, FRANCE

## Abstract

Hypospadias (H) is a common birth defect affecting the male urinary tract. It has been suggested that exposure to endocrine disrupting chemicals might increase the risk of H by altering urethral development. However, whether H risk is increased in places heavily exposed to agricultural pesticides, such as vineyards, remains debated and difficult to ascertain. The objective of the work is to test the possible association of H with residential proximity to vineyards. Residential address at birth of 8,766 H cases born 1980–2011 was taken from 17 specialized surgery centers. The geographical distribution of vineyards was obtained from the European Land Parcel Identification System (LPIS) and the distance of address to the nearest vineyard was computed. A first estimate of the variation of H relative risk with distance to vineyards was obtained using as controls 13,105 cryptorchidism (C) cases operated during the same period in the same centers. A separate estimate was obtained from a case-control study using “virtual controls” (VC) defined as points of the map sampled to match the demographic distribution of births within the recruitment territories of the study centers. Non-exposed patients were defined as those with a residence between 5,000 and 10,000 m from the closest vineyard. The residential distance to vineyard was smaller for H than for C cases (p<10^−4^). We found 42/8766 H cases (0.48%) and 50/13,105 C cases (0.38%) born to mothers living within 20 m of a vineyard. The odds ratios for H were 2.48 (CI: 1.0 to 5.1) and 2.4 (CI: 1.3 to 4.4), vs C or vs VC, respectively, when pregnant mothers lived 10–20 m from a vineyard. In conclusion, our study supports that children born to mothers living close to a vineyard have a two-fold increased risk of H. For environmental research, the use of VC provides an alternative to classical case control technique.

## Introduction

Hypospadias (H) is a defect in the urethral development occurring between the 8^th^ and the 16^th^ week of pregnancy causing the meatus to be ill-located on the ventral side of the penis. While H is a frequent congenital malformation that needs early correction by urethroplasty [1], its epidemiology has remained imprecise. Since the early 2000s, H incidence has shown large and unexplained differences across populations and time periods [2–5]. In 2003, H incidence was estimated to be 8/10,000 live male births in the US [2] and 18.6/ 10,000 in a study of 23 European national registries [3]. H incidence had increased until 1999 according to 36 years of Swedish data, [6], but no variation was observed between 2004 and 2010 across European registries [3].

Since less than 1% of H cases are due to rare monogenic syndromes, the vast majority are still called “idiopathic” and are presumably due to unknown developmental interaction between gene variants and environmental factors. Genetic predisposition is important, as shown by the 12 to 20-fold augmentation of H) among first-degree relatives [7]. Genome-wide studies have identified a few low-risk genomic variants associated with H [8], while epigenetic studies have started to explore the association of H with DNA methylation [9, 10]. The contribution of environmental factors is likely to be prominent, but has been difficult to assess. A link of H with decreasing male fertility suggests the existence of a “testicular dysgenetic syndrome” that has emerged in developed countries [11].

Agricultural pesticides—prominently suspected endocrine disruptors—remain the main environmental suspects, but studies have yielded variable results across countries, regions and time periods [12], Case-control studies of parental occupation did not show an increased risk of H in sons of women working in farming and gardening [13–15], but none had specifically focused on people working in vineyards.

A direct way to assess the exposures of cases and controls to agricultural pesticides is offered by Geographical Information Systems (GIS) and the increasing availability of environmental data bases informing on land use [16]. For example, the comparison of exposures of 354 cases with 727 controls with regard to 38 pesticides within 500 m of pregnant mother’s address in Arkansas found a slight association of H with diclofop-methyl (OR = 1.08), and surprisingly a stronger protective effect of other pesticides (e.g. permethrin: OR = 0.37) [17]. Among 304,906 singletons born in North Carolina, the estimated amounts of pesticides used within 500 meters of maternal residence yielded only a marginal association with H in 856 cases [18]. In France, an increase of H incidence at the proximity of Montpellier vineyards has been debated [19] [20]. The fact that many vineyards are located near houses in many countries calls for robust epidemiological evidence based on reliable controls. Notably, a reliable control is”someone who–if he had developed the disease- would have been recruited among the cases of the study” [21].

Surgical centers do not usually record epidemiological information on the exposure history of their patients, but all of them record basic demographic information for billing purposes, including mother’s address at childbirth. This low-cost hospital information crossed with available geographic environmental databases was used for the current study. We have used two independent approaches to define appropriate controls to which the cases should be compared. First we used as controls the cases of C recruited in the same network of surgical centers than those used for H. These C patients could be considered as H controls since all included cases of C had a normal penis and meatus, while none of the studied H cases had mal-descended testes. We did a second independent case-control analysis, where controls were “virtual” controls (VC) defined through an algorithmic approach described below.

## Material and methods

### Patients

To be included in this study, H cases were the clinically significant cases that had reconstructive surgery in the line of the current practice [22], and C cases had to have orchidopexy [23]. Minimal glandular hypospadias cases that are considered clinically not significant, thus not operated, were not part of our study. Under coordination by L.Esterle and Pr P.Mouriquand (Lyon), the following 17 French centers of urological pediatric surgery (Table 1) collected retrospectively the date of birth and address at birth of the parents of all boys born after January 1st 1980 who had H (coded as ICD Q54) and C (coded as ICD Q53): Angers (Pr G. Podevin), Bordeaux (Pr B. Frémond), Caen (Pr P. Ravasse), Colmar (Pr S. Geiss), Grenoble (Pr B. Boillot), Lyon (Pr P. Mouriquand), Marseille and Toulon (Pr P. Alessandrini), Vandoeuvre-lès-Nancy (Pr JL. Lemelle), Nantes (Pr MD. Leclair), Reims (Pr PJ. Lefebvre), Rennes (Pr E. Dobremez), Saint Étienne (Pr F. Varlet), Strasbourg (Pr R. Moog), Saint Vincent de Paul at Paris (Pr F. Bargy), Toulouse (Pr J. Moscovici), Valence (Pr B.Defauw).

**Table 1:** Numbers (and %) of cases and controls analyzed in each collaborative center.

Center	H Cases	C Cases	Virtual Controls
*Total*	*8766*	*13105*	*43830*
Angers	57 (0.7)	460 (3.5)	285 (0.7)
Bordeaux	232 (2.6)	249 (1.9)	1160 (2.6)
Caen	554 (6.3)	1266 (9.7)	2770 (6.3)
Colmar	373 (4.3)	396 (3.0)	1865 (4.3)
Grenoble	148 (1.7)	51 (0.4)	740 (1.7)
Lyon	1688 (19.3)	2787 (21.3)	8440 (19.3)
Marseille	353 (4.0)	152 (1.2)	1765 (4.0)
Nancy	267 (3.0)	624 (4.8)	1335 (3.0)
Nantes	871 (9.9)	1102 (8.4)	4355 (9.9)
Reims	446 (5.1)	826 (6.3)	2230 (5.1)
Rennes	664 (7.6)	1100 (8.4)	3320 (7.6)
Saint-Etienne	361 (4.1)	698 (5.3)	1805 (4.1)
Strasbourg	924 (10.5)	1008 (7.7)	4620 (10.5)
Saint-Vincent Paris	463 (5.3)	219 (1.7)	2315 (5.3)
Toulon	0 (0.0)	196 (1.5)	0 (0.0)
Toulouse	1072 (12.2)	1633 (12.5)	5360 (12.2)
Valence	293 (3.3)	338 (2.6)	1465 (3.3)

The two sets of controls are cases with cryptorchidism (C) and “virtual controls” (see Material and methods).

All children operated for hypospadias in the University hospitals participating to this study had all been carefully examined at a pediatric endocrinology unit belonging to the Centre National for Disorders of Sex Development (DSD). Cases resulting from a genetic disorder of sex differentiation or hypogonadotropic hypogonadism were excluded. At total, 8,766 H and 13,105 C cases were recorded by May 2013. (see Table 1). We computed the distance of each case to the nearest vineyard (see below), and constituted the database which is analyzed in this paper.

**Fig 1:**
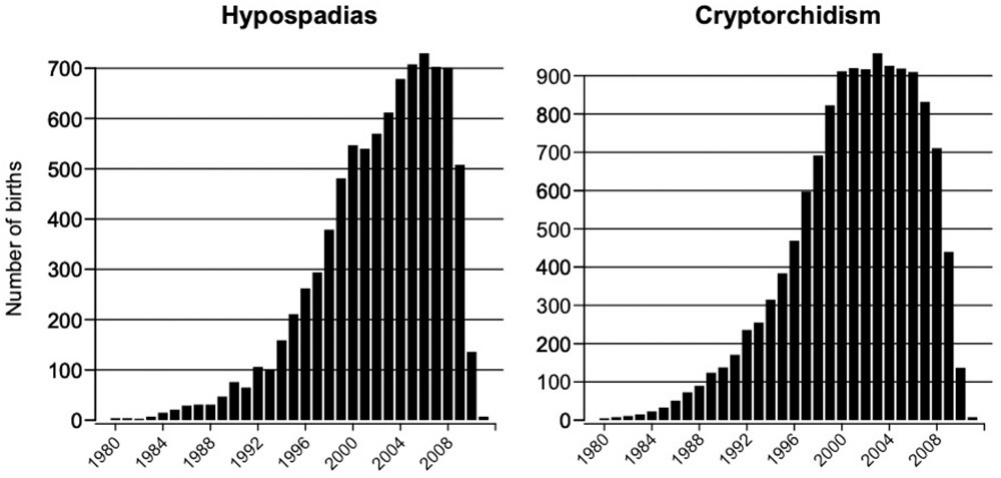
Distribution of the year of birth distribution cases (H: Left, C: Right).

The distribution of the year of birth of the H and C cases is shown in Fig 1.

### Ethics, data confidentiality

The research protocol was approved by the Ethics committee of Ile de France (DC-2009-1041). The parents of children provided written informed consent. To protect the confidentiality and data of participants, all were assigned a unique identification number without identifying information. The security conditions of the database received the agreement of the “national information science and liberties commission” (ref CNIL 912529). Under this agreement, CNIL can allow external researchers to get access to the original databases. Requests must be sent to the board of the HCFCG (HypoCrypto French Collaborative Group, Inserm, Batiment Pincus, Hopital de Bicêtre, 94276- Le Kremlin Bicêtre).

### Assessment of the residential proximity of people to vineyards

We used the Land Parcel Identification System (LPIS) to assess the residential proximity of the homes of the children to vineyards. The origin of LPIS is a decision of the European Union Council Regulations in 2003 to create an information system on the land use of European agricultural parcels.

In LPIS, an agricultural parcel is defined as a continuous area of land on which an individual farmer cultivates a single crop. The data is encoded using the shapefile format, which is a GIS file format describing vector features such as points, lines, and polygons. Polygons are used in order to specify the borders of the parcels. The geographic projection system used to specify the edges of these polygons is Lambert-93. Each item element in shapefile has attributes. The attribute of a polygon is a number that identifies the crop being grown on that parcel. LPIS information was made available for years 2010 to 2014. We used in this work the 2012 database that was freely downloadable from the French data.gouv.fr web portal (https://www.data.gouv.fr/fr/datasets/registre-parcellaire-graphique-2012-contours-des-ilots-culturaux-et-leur-groupe-de-cultures-majorita/). At total the current study is based on the 340,336 LPIS parcels with vineyards, for a total surface of 381,395 Ha.

The exposure metric that we use in this work is the individual’s distance to the nearest parcel of vineyard. In practice, we had to calculate these distances for more than 50,000 individuals (cases plus controls) to 340,336 parcels of vineyard. Each parcel was modeled as a polygon. To avoid calculating exact distances to polygons that are sufficiently far away for having any chance to induce a risk, we chose a cutoff at 10,000 meters and pooled all distances greater than 10,000 m in a single class.

### Statistical analysis of the risk associated with distance to the nearest vineyard

Two analyses were performed. The first one was based on patients whose home was located in three large classes of distances to a vineyard: 0–100 m, 100–200 m and 200–500 m. The second analysis studied the risk at distances of less than 50 m from a vineyard, despite the fact that only 82 cases were from homes located there. The three classes of “small” distances studied were 0–10 m, 10–20 m and 20–50 m.

In both analyses we studied H risk in two independent case-control studies, taking the 5,000–10,000 m class of distance as reference. In the first analysis, we compared the distances to the nearest vineyard in the two groups of H and C cases. For this comparison, we computed the variation of the odds ratio of the H risk, by reference to the C risk, in the different zones of distance to a vineyard, taking the 5,000 to 10,000 m distance as the reference.

In the second analysis, we computed the variation of the odds ratio (OR) for the H risk of people living within zones close to vineyards, taking as reference the risk of children born to mothers living between 5,000 and 10,000 m, i.e. roughly 21% of the patients (Table 2). The specificity of our approach is that the controls of this case-control study were “virtual controls” (VC). A “virtual control” is a geographical point of the map which represents a real physical person that would have been chosen as control, if it was practically possible to sample randomly a subject within the same source population as the “case”. We modeled the source population of each hospital as composed of the population living in the circle around this center, in which there was a proportion p of the cases. In the analyses presented here, p was taken at 80%. The algorithm used to sample the VC is the PPS algorithm implemented by Jack G. Gambino as an R package (PPS stands for probability proportional to size). https://rdrr.io/cran/pps/. This algorithm allows to sample people in a territory proportionally to the local density of population. The French National Institute of Statistics (INSEE) 2009 database (https://www.insee.fr/fr/statistiques/2520034) provided us with an estimate the population density. This database provides the 2009 French density of population in each age-class within a 200 m resolution grid. From this 2009 information, we can estimate the spatial distribution of children born, say, in 1995: assuming that mobility was low (which is the case in France), this distribution is simply the distribution of the children aged 14 (= 2009–1995) in the 2009 grid. This spatial distribution is then used to randomly chose–thanks to PPS- a cell within all the cells that constitute the source population. The VC is then set at the center of the 200m x 200m cell. We took 5 virtual controls per case by repeating the algorithm 5 times.

**Table 2:** Proportions of hypospadias and cryptorchidism in different classes of distance to the nearest vineyard.

Distance (m)	Hypospadias (%)	Cryptorchidism (%)
[0, 10]	0.27	0.30
[10, 20]	0.21	0.08
[20, 50]	0.46	0.32
[50, 100]	0.78	0.73
[100, 200]	1.65	1.46
[200, 500]	3.18	3.20
[500, 1 000]	4.29	3.72
[1 000, 5 000]	26.24	24.18
[5 000, 10 000]	21.70	24.24
>10 000	41.23	41.77

### Socioeconomic environment

The 2009 version of the geographic information system of the French National Institute of Statistics (INSEE) (https://www.insee.fr/fr/statistiques/2520034) provided us with an estimate of the density of population, by age class, in each square of a 200m x 200m grid, **repeat** and an estimate of the local socioeconomic environment by using the Townsend deprivation index (TDI) [24] at the place of birth. A higher TDI score implies more severe social deprivation.

### Statistical packages

The odds ratios quantifying the association of disease and distance to the nearest vineyard were estimated using logistic regression adjusted for the coordinating center, to account for difference in the distance distribution between centers. Additional analyses were adjusted for TDI. Subgroup analyses by birth period were also carried out. Analyses were carried out using the R statistical software version 3.6.3, with the glm function of the stats package.

## Results

The distribution of the distance of the children affected with H or C to the nearest vineyard is shown in Table 2. The two distributions were significantly different (chi square = 38, 9 df, p<10^−4^). H cases were more frequent than C in all classes of distances below 5,000 m to vineyards, with the exception of those located between 0 and 10 m.

We first analyzed wide classes of distance (0–100 m, 100–200 m, 200–500 m, 500–1000 m), where the number of H patients was large (between 145 and 376). We found that odds ratios were > 1 in all these classes of distance, and were significantly > 1 in the 0–100m class (Table 3) when controls were patients with cryptorchidism (Column 1 of Table 3), and when controls were “virtual controls” after adjustment on the Townsend index (column 2b of Table 3).

**Table 3:** Odds ratios, with 95% confidence intervals, of the risk of hypospadias by classes of distance to the closest vineyard. (large distances).

Distance	1: Controls with cryptorchidism	2a: Virtual controls	2b: Virtual controls adjusted on TDI
0–100 m	1.26 (1.00 to 1.59)	1.11 (0.91to 1.37)	1.26 (1.02 to 1.52)
100–200 m	1.25 (0.99 to 1.57)	1.22 (1.00 to 1.50)	1.37 (1.11 to 1.68)
200–500 m	1.09 (0.92 to 1.29)	1.00 (0.86 to 1.16)	1.10 (0.95 to 1.28)

Column 1: the controls are C cases. Columns 2a and 2b: the controls are “virtual controls”. Column 2b shows the odds ratios after adjustment on the Townsend Deprivation Index (TDI).

In the second part of the analysis, we studied the patients whose houses were located at close distances of vineyards (less than 50 m), although their numbers were small (see Table 2). Again, we estimated the risk of H possibly associated with a short distance from the nearest vineyard by computing the odds ratios of H patients versus C patients, and the odds ratios of patients versus VC. The two analyses gave comparable results. The odds ratio of H patients in the 0–50 m was significantly > 1 (column 1, where controls are patients with C and column 2b, where controls were “virtual controls”). When we focused on classes of addresses at very short distances of a vineyard (second part of Table 4), we found that the odds ratio of H patients belonging to a home located between 10 and 20 m from the closest vineyard was significantly > 1 (p = 0.029 when controls are C patients, p = 0.007 when controls are “virtual controls”). The results were comparable after adjustment on the Townsend Index.

**Table 4:** Odds ratios, with 95% confidence intervals, of the risk of hypospadias by classes of distance to the closest vineyard. (short distances).

Distance	1: Controls with cryptorchidism	2a: Virtual controls	2b: Virtual controls adjusted on TDI
0–50 m	1.40 (1.02 to 1.92)	1.23 (0.93 to 1.61)	1.38 (1.05 to 1.82)
0–10 m	0.97 (0.57 to 1.66)	0.84 (0.51 to 1.39)	0.96 (0.58 to 1.58)
10–20 m	2.48 (1.10 to 5.62)	2.33 (1.26 to 4.29)	2.60 (1.41 to 4.80)
20–50 m	1.51 (0.97 to 2.36)	1.26 (0.87 to 1.84)	1.42 (0.98 to 2.07)

Column 1: the controls are C cases. Columns 2a and 2b: the controls are “virtual controls”. Column 2a: all H cases. Column 2b: odds ratios after adjustment on the Townsend Deprivation Index (TDI).

## Discussion-conclusion

Common “idiopathic” H results from unknown mechanisms, among which endocrine disruptors are suspected to interfere with gonadal and genital development of the male fetus.

We found in this work that the risk of H was higher in children born to mothers living close to a vineyard.

The strength of our observation is that it was achieved through two independent methodological approaches. Both methods guarantee that controls were from the same hospital recruitment territory than cases. This avoids the bias of controls made up of children with common pediatric diseases that usually live close to the center, whereas cases of H come from far away for a very specialized intervention. Such bias could create the false impression that patients from rural areas would be more at risk than those from urban areas. The controls who were used in the two approaches match the ideal definition of what must be a control group: "individuals who, if they had had the disease (H), would have been recruited in the same centers as the (H) cases were" [21]. In the first approach, the controls were children with C and no H having been operated in the same clinical centers as H. A few studies found a possible positive association between C and exposure to pesticides. However, even if this link is true, it would not invalidate our results. Indeed, the consequence of such a link would be that the “true” odd-ratio is even greater than the one we found. In the second approach, the “virtual controls” were not real persons. However the algorithm that we used guarantees that they are identical to the population of children that would be sampled in the “territory” of the cases. To realize this, we modeled the recruitment “territory” of an hospital as the population living in a virtual circle around this hospital containing a proportion p = 80% of the cases. We also performed the analyses using other models to define hospital territory, and obtained similar results.

The increased H prevalence at short distances from vineyards is an indirect indication supporting, but not proving, a harmful effect of pesticides since our study did not qualify nor quantify the loads and nature of pesticides spread in the vineyards. Indeed, this was an impossible task given the 31-year duration of our H case collection. Not only pesticides, such as fungicides, insecticides, herbicides, bactericides, rodenticides, fumigants, but also fertilizers and other toxic chemicals were and still are commonly used for viticulture. During the long studied period providing our data, the complexity, variety and dynamic mix of chemicals used in vineyards were considerable, and no database was available to quantify precisely their nature and load. The found association of vineyard proximity with H risk could also be due to other environmental factors present close to vineyards. Known endocrine disruptors commonly used in viticulture at the time of the study are vinclozolin, procymidone, and linuron, all forbidden since 2007–2008, but still present in some soils close to vines. The fungicide vinclozolin and its metabolites act as androgen receptor antagonists [25]. Procymidone is another anti-androgenic fungicide that can induce hypospadias in rodents [26]. Linuron, a widely used herbicide, is a weaker androgen receptor antagonist than procymidone and vinclozolin [27]. Nitrates are another category of inorganic pollutants that can disrupt gonadal steroidogenesis [28, 29]. It was interesting in this respect to search whether the association of H with vineyard proximity was stronger in the years before 1995, but the small number of cases did not allow a reliable analysis.

Agricultural substances may diffuse via surface runoff, leaching to field drains, and atmospheric spray drift. Pesticide drift may occur during ground application and after it, since applied substances have different volatility and local persistence. Our choice of focusing on the narrow zone bordering vineyards was influenced by methods used to apply pesticides, which never include aerial sources such as in the US but only land-based devices.

Vineyards are a privileged place for pesticide spraying, undoubtedly one of the most important of all French agriculture. A weakness of our study is that it did not get occupational data for the mothers of cases, although one can expect that a much higher percentage of those living in the vicinity of vineyards are occupationally involved in wine producing activity, thus possibly exposed to pesticide stocks, bisphenol A, phthalates, or other potential endocrine disruptors [30].

It must be noted that the possible risk associated to being a fœtus developed within a short distance of a vineyard concerns only a small proportion of the H cases: H cases born in a house at less than 20 m from a vineyard represent only 0.5% of the total number of H patients (those born at less than 50 m are 1%, and at less than 100 m are 1.8% of the overall H prevalence in France).

The VC approach makes the best possible use, at almost no cost, of the huge data resource that is sleeping in hospitals instead of serving epidemiology. It does not require filling questionnaires or track families to get controls, but instead relies on the basic medical information system. In addition, the method avoids some selection bias, in that we did not have to obtain subjects’ consent to participate, as we used only information in the public domain. For all of these reasons, we believe that the VC method could be used increasingly for well-defined diseases using more detailed, and frequently refreshed environmental databases. A limitation of the method is that VC cannot be studied to compare biological exposome with cases, including pesticide and metabolite concentrations in biological fluids or cells [31].

In conclusion, we have found a significant statistical link between the occurrence of H and the residence of pregnant mothers close to vineyards. However we are conscious that generalizations that focus on ‘positive’ findings rather than more comprehensive views that incorporate null findings and study limitations should be avoided. Future environmental research will have to account for the changing nature and loads of pesticides in modern viticulture. This could be done with the method presented, if high-resolution public reliable databases on the geographical use of different pollutants become available to public health research. In addition, further identification of precise hormone-disruptors will require a deep, systematic and specific chemical investigation of vineyards and their close neighborhood.

More generally, the technique of “virtual controls” that was used in this work can be applied to search for candidate environmental factors in fetal diseases where the only available information is the location of the patient at birth.

## Supporting information

S1 File (DOCX)
