# Supplementary material for: Exploring the risk of hypospadias in children born from mothers living close to a vineyard
Source: PLoS One. 2021 Apr 15;16(4):e0249800. doi: 10.1371/journal.pone.0249800 (PMC8049337; doi:10.1371/journal.pone.0249800)
Supplement: S1 File — (DOCX) [file pone.0249800.s001.docx]

**Exploring the risk of hypospadias in children born from mothers living close to a vineyard: supporting information.**

Pierre Bougnères^1^, Raphael Porcher^2^, Laure Esterle^1^, David Baker^1^, Adrien de la Vaissière^1^, Sofia Meurisse^1^, Sophie Valtat^1^, Anne-Laure Castell**^1^**, Pierre Mouriquand^3^, Alain-Jacques Valleron^1^

## List of contributors to the project.

| Centre | Hôpital | Investigateurs | telephone | COURRIEL |
| --- | --- | --- | --- | --- |
|  | CHU AMIENS  Service de chirurgie Pédiatrique  4 place Victor Pauchet  80034 AMIENS CEDEX | dr elodie haraux | 06 71 19 61 00 | haraux.elodie@chu-amiens.fr |
|  | CHU ANGERS  CHIRURGIE PeDIATRIQUE  49033 Angers cedex 01 | PR GUILLAUME PODEVIN | 02 41 35 49 13 |  |
|  | CH AVIGNON  Service de Chirurgie Infantile  305 rue Raoul Follereau  84 000 Avignon | DR GIANLUCA DE LUCA | 04 32 75 36 90 | jldeluca@ch-avignon.fr |
|  | Chu besançon-hôpital saint jacques  Chirugie infantile  25030 besancon cedex | Pr Didier aubert | 03 81 21 82 21 | [daubert@chu-besancon.fr](mailto:daubert@chu-besancon.fr) |
|  | CHU Pellegrin-Hôpital des Enfants  CHIrurgie pédiatrique  33000 BORDEAUX | Pr Eric Dobremez | 05 56 79 56 23 | [eric.dobremez@chu-bordeaux.fr](mailto:eric.dobremez@chu-bordeaux.fr) |
|  | CHU Morvan  Chirurgie Pédiatrique  29609 Brest Cedex | Dr Philine de VRIES | 02 98 22 39 26 | [philine.devries@chu-brest.fr](mailto:philine.devries@chu-brest.fr) |
|  | CHU Côte de Nacre  Chirurgie Pédiatrique  14000 CAEN | Pr Philippe RAVASSE | 02 31 06 44 89 | [ravasse-p@chu-caen.fr](mailto:ravasse-p@chu-caen.fr) |
|  | CME le Parc  46 rue du Stauffen  Chirurgie Pédiatrique 68024 Colmar cedex | Dr Stephan GEISS | 03 89 12 60 82 | [stephan.geiss@ch-colmar.fr](mailto:stephan.geiss@ch-colmar.fr) |
|  | CHU Grenoble Hôpital A MICHALLON  chirurgie pédiatrique 38043 GRENOBLE CEDEX 9 | Dr Bernard Boillot | 04 76 76 59 19 | [bboillot@chu-grenoble.fr](mailto:jfdyon@chu-grenoble.fr) |
|  | CHU LE HAVRE  Service de Chirurgie Infantile - PFME  76083 Le Havre CEDEX | Dr Brigitte Hugueny-Pech de Laclause | 02 32 73 42 30 | [brigitte.hugueny@ch-havre.fr](mailto:brigitte.hugueny@ch-havre.fr) |
|  | CH Lyon Hôpital debrousse  chirurgie pédiatrique viscerale et urologiquie  69677 Bron | Pr Pierre Mouriquand / Dr daniela gorduza | 04 72 38 56 50 | [pierre.mouriquand@chu-lyon.fr](mailto:pierre.mouriquand@chu-lyon.fr) |
|  | CHU Lille Hôpital Jeanne de Flandres  chirurgie pediatrique  59037 LILLE | Pr Remi besson | 03 20 44 57 15 | [remi.besson@chru-lille.fr](mailto:remi.besson@chru-lille.fr) |
|  | Hôpital Lapeyronie, CHU de montpellier  service de Chirurgie viscerale et urologique pediatrique  371 avenue giraud 34295 Montpellier cedex 5 | Pr Nicolas Kalfa | 04 67 33 87 84 | [nicolaskalfa@gmail.com](mailto:nicolaskalfa@gmail.com) |
|  | CHU NANCY-HÔPITAL DE BRABOIS  Chirurgie urologique  54511 VANDOEUVRE-LES-NANCY | Pr Michel Schmitt | 03 83 15 46 74 | [m.schmitt@chu-nancy.fr](mailto:m.schmitt@chu-nancy.fr) |
|  | CHU Nantes -hôpital mère-enfant  Chirurgie infantile  44093 Nantes Cedex 1 | Pr Marc david leclair | 02 40 08 35 85  06 77 16 87 88 | [marcdavid.leclair@chu-nantes.fr](mailto:marcdavid.leclair@chu-nantes.fr) |
|  | HOPITAL SAINT VINCENT DE PAUL – COCHIN  rue Saint jacques  75014 paris | Pr Frédéric BARGY | 01 44 49 41 54 | frederic.bargy@nck.aphp.fr |
|  | Hôpital Robert-Debré  Chirurgie infantile a orientation viscerale  75935 PARIS CEDEX 19 | [Pr Alaa EL GHONEIMI](http://www.aphp.fr/index.php?module=offredesoins&action=afficherMedecin&vue=ods_m_medecin&hopital=070&service=0007&nom=EL%20GHONEIMI&prenom=Alaa) | 01 40 03 21 59 | [alaa.elghoneimi@rdb.aphp.fr](mailto:alaa.elghoneimi@rdb.aphp.fr) |
|  | CHU Reims  Chirurgie Pédiatrique  47 rue Cognac Jay  51092 Reims cedex | Dr Francis Lefebvre | 03 26 78 36 15 | [flefebvre@chu-reims.fr](mailto:flefebvre@chu-reims.fr) |
|  | CHU Rennes-HOPITAL SUD  chirurgie pediatrique  35033 RENNES cedex 9 | Pr Benjamin FREMOND | 02 99 28 43 21 | [benjamin.fremond@chu-](mailto:benjamin.fremond@chu-)rennes.fr |
|  | CHU ROUEN  CLINIQUE CHIRURGICALE INFANTILE – PAVILLON DE PEDIATRIE  1 RUE DE GERMONT  76031 ROUEN CEDEX | Pr Agnès LIARD-ZMUDA | 02 32 88 81 61 | agnes.liard-zmuda@chu-rouen.fr |
|  | CHU SAINT ETIENNE - Hopital Nord  chirurgie pediatrique  42277 ST PRIEST EN JAREZ CEDEX | Pr François VARLET | 04 77 82 80 37 | [francois.varlet@chu-st-etienne.fr](mailto:francois.varlet@chu-st-etienne.fr) |
|  | CHrU STRAsbourg – Hôpital Hautepierre  Chrirugie pediatrique  67098 strasbourg | dr raphael moog | 03 88 12 73 05  (ligne directe) | [raphael.moog@chru-strasbourg.fr](mailto:raphael.moog@chru-strasbourg.fr) |
|  | CH TROYES  Service d’Urologie  101 avenue Anatole France  10003 Troyes | Dr bruno leroux | 03 25 75 32 85 | doc.leroux@wanadoo.fr |
|  | CHU Toulouse  pediatrie chirurgie viscerale  31059 Toulouse cedex 9 | Pr Jacques Moscovici | 05-34-55-85-13 | [moscovici.j@chu-toulouse.fr](mailto:moscovici.j@chu-toulouse.fr) |
|  | CHU CLOCHEVILLE  Chirurgie pediatrique 37044 TOURS cedex 9 | PR Hubert Lardy | 02 47 47 38 20  02 47 47 37 41 | [hubert.lardy@univ-tours.fr](mailto:hubert.lardy@univ-tours.fr) |
|  | CHI TOULON-Hôpital Font-Pré  PEDIATRIE  83056 Toulon CEDEx | PR PIERRE ALESSANDRINI | 04 94 61 60 06 | [pierre-jean.alessandrini@mail.ap-hm.fr](mailto:pierre-jean.alessandrini@mail.ap-hm.fr) |
|  | CH Valence  Chirurgie pediatrique  26953 Valence | DR Benoit Defauw | 04 75 75 72 16 | [bdefauw@ch-valence.fr](mailto:bdefauw@ch-valence.fr) |
|  | AP-HM hopital nord  Chemin des Bourrely, 13015 Marseille | PR PIERRE ALESSANDRINI | 06 08 16 77 42 | [pierre-jean.alessandrini@mail.ap-hm.fr](mailto:pierre-jean.alessandrini@mail.ap-hm.fr) |
